# DYRK1A genetic variants are not linked to Alzheimer's disease in a Spanish case-control cohort

**DOI:** 10.1186/1471-2350-10-129

**Published:** 2009-12-08

**Authors:** José Luis Vázquez-Higuera, Pascual Sánchez-Juan, Eloy Rodríguez-Rodríguez, Ignacio Mateo, Ana Pozueta, Ana Frank, Isabel Sastre, Fernando Valdivieso, José Berciano, María J Bullido, Onofre Combarros

**Affiliations:** 1 Neurology Service and CIBERNED, "Marqués de Valdecilla" University Hospital (University of Cantabria), Santander, Spain; 2 Neurology Service and CIBERNED, Hospital Universitario La Paz (UAM), Madrid, Spain; 3 Molecular Biology Department and CIBERNED, Centro de Biología Molecular Severo Ochoa (CSIC-UAM), Madrid, Spain

## Abstract

**Background:**

As dual-specificity tyrosine phosphorylation-regulated kinase 1A (DYRK1A) has been implicated in the abnormal hyperphosphorylation of tau in Alzheimer's disease (AD) brain, and the development of neurofibrillary tangles, we examined the contribution of this gene to the susceptibility for AD.

**Methods:**

We examined genetic variations of DYRK1A by genotyping haplotype tagging SNPs (htSNPs) (rs11701483, rs2835740, rs1137600, rs2835761, rs2835762, rs2154545 and rs8132976) in a group of 634 Spanish AD cases and 733 controls.

**Results:**

There were no differences in the genotypic, allelic or haplotypic distributions between cases and controls in the overall analysis or after stratification by APOE ε4 allele.

**Conclusion:**

Our negative findings in the Spanish population argue against the hypothesis that DYRK1A genetic variations are causally related to AD risk. Still, additional studies using different sets of patients and control subjects deserve further attention, since supporting evidence for association between DYRK1A gene and AD risk in the Japanese population exists.

## Background

Abnormal tau hyperphosphorylation has been suggested as being one of the central events in the development of neurofibrillary tangles (NFTs), which are one of the characteristic neuropathological lesions found in Alzheimer's disease (AD) brains [1]. Dual-specificity tyrosine phosphorylation-regulated kinase 1A (DYRK1A) phosphorylates tau in vitro at the Thr212 residue [2], which is hyperphosphorylated in AD brains, and a significant increase in the amount of phosphor-Thr212-tau is also found in the brains of transgenic mice that overexpress human DYRK1A [3]. In addition, transgenic mice bearing a triple tau mutation and expressing hyperphosphorylated tau in neurons of the hippocampus and neocortex show increased expression of DYRK1A in individual neurons in the same regions [4]. Moreover, DYRK1A accumulates in NFTs in brains of subjects with sporadic AD and in subjects with trisomy of chromosome 21 and Down syndrome (DS) [5]. The increase dosage of DYRK1A in DS brain due to trisomy of chromosome 21 correlates to an increase in three microtubule-binding domain repeats-tau level [6], which on abnormal hyperphosphorylation and aggregation of tau results in neurofibrillary degeneration [7]. All this data postulates a role for DYRK1A as an interesting genetic target for association analysis of AD. Although genetic markers of the DYRK1A region were not found associated to AD in recent genome-wide association studies [8-11], Kimura et al. [12] scanned througt chromosome 21 to assess genetic associations with late-onset AD and found that DYRK1A showed the highest significant association with AD risk in the Japanese population. In addition, these authors suggested that DYRK1A could be a key molecule bridging between β-amyloid production and tau phosphorylation in AD [12]. In this report we sought to replicate this genetic association in the Spanish population.

## Methods

The study included 634 AD patients (65% women; mean age at study 75.9 years; SD 8.0; range 61-109 years; mean age at onset 72.8 years; SD 7.9; range 60-108 years) who met NINCDS/ADRDA criteria for probable AD [13]. All AD cases were defined as sporadic because their family history did not mention any first-degree relative with dementia. AD patients were recruited from the Departments of Neurology of University Hospital "Marqués de Valdecilla" (Santander, Spain), and Hospital "La Paz" (Madrid, Spain). The large majority of patients were living in the community and had been referred by their general practitioner; few had been admitted from hospital wards or nursing home facilities. Control subjects were 733 unrelated individuals (65% women; mean age 78.7 years; SD 9.4; range 60-104 years) randomly selected from nursing homes. These subjects had complete neurologic and medical examinations that showed that they were free of significant illness and had Mini Mental State Examination scores of 28 or more (corrected for age), which were verified by at least one subsequent annual following-up assessment. The controls arose from the same base population as the cases. The AD and control samples were Caucasians originating from a limited geographical area in northern Spain (Santander) and from the central area of Spain (Madrid).

**Figure 1:**
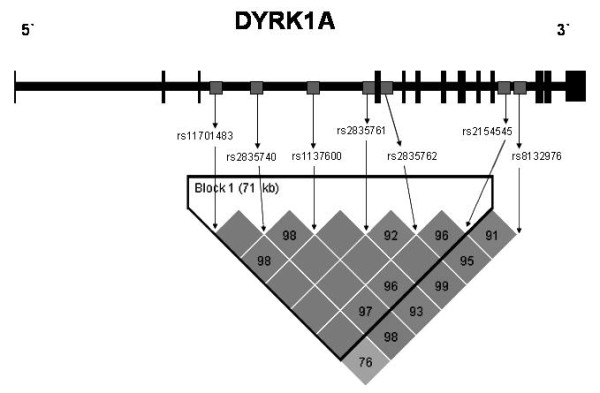
**Genomic structure and relative location of studied haplotype tagging SNPs (indicated by grey boxes) in the DYRK1A gene**. Lines represent the introns between exons (black boxes). Pairwise linkage disequilibrium (LD) patterns between the 7 htSNPs formed one haplotype block (numbers in box represent D' values, and the intensity of the color is proportional to the strength of the LD).

Blood samples were taken after written informed consent had been obtained from the subjects or their representatives. The study was approved by the ethical committees of the University Hospital "Marqués de Valdecilla" and the Hospital "La Paz". Genotyping of DYRK1A (rs11701483, rs2835740, rs1137600, rs2835761, rs2835762, rs2154545 and rs8132976) polymorphism was performed by a Taq-Man single-nucleotide-polymorphism assay (Applied Biosystems, Warrington, Cheshire, UK) and an ABI PRISM 7000 or 7900HT sequence detection systems (Applied Biosystems). We used data from the HapMap project http://www.hapmap.org to select the 7 htSNPs capturing 86% of DYRK1A genetic variability in Caucasians. SNPs were chosen among those with minor allele frequencies ≥ 5% using Haploview v3.2 software http://www.broad.mit.edu/mpg/haploview with an r^2 ^threshold of 0.8. The location of SNPs in DYRK1A gene used in the present study is described in Figure 1. APOE genotyping was performed by amplification of the 4th exon of the APOE gene by PCR with biotinylated primers, followed by reverse hybridization on nitrocellulose strips, using the INNO-LIPA ApoE assay (Innogenetics NV, Ghent, Belgium), or by HhaI restriction analysis.

Hardy-Weinberg equilibrium (HWE) was calculated for the 7 htSNPs genotypes in the control population using Pearson's χ^2 ^statistics. We assessed pairwise linkage disequilibrium (LD) between the 7 htSNPs by D' and r^2 ^statistics. Haplotype reconstruction and their frequencies in cases and controls were estimated by an expectation-maximization algorithm. Pearson's χ^2 ^statistics were performed to compare allele distribution of the patients and control for each htSNP. Haplotype frequencies were also assessed using Pearson's χ^2 ^using Haploview 3.32 software http://www.broad.mit.edu/mpg/haploview. Rare haplotypes (total frequency < 0.05) were excluded from the analysis.

## Results

In control groups, no deviations from Hardy-Weinberg equilibrium were found for any of the 7 htSNPs. As shown in Table 1, the distribution of the allele and genotype frequencies of the DYRK1A htSNPs did not differ significantly between either un-stratified or APOE-stratified AD and control groups. Figure 1 shows the pattern of pair wise LD, measured in terms of D' coefficient, between the 7 chosen htSNPs. One block was found consisting of SNPs rs11701483, rs2835740, rs1137600, rs2835761, rs2835762 and rs2154545. When we estimated the haplotype frequencies in this haplotype block, we found that the haplotype distribution did not differ significantly between AD cases and controls (Table 2). There were no major differences in allele, genotype or haplotype frequencies of DYRK1A polymorphisms in our total sample associated to either age or gender subgroups (data not shown).

**Table 1:** Distribution of DYRK1A polymorphisms in patients and controls stratified by APOE ε4 allele

DYRK1A polymorphism		APOE ε4 allele noncarriers		APOE ε4 allele carriers		Total sample	
			
		Patients	Controls	Patients	Controls	Patients	Controls
rs11701483	AA	226 (0.78)	486 (0.79)	273 (0.83)	85 (0.74)	499 (0.81)	571 (0.79)
	AG	58 (0.20)	119 (0.20)	54 (0.16)	27 (0.24)	112 (0.18)	146 (0.20)
	GG	6 (0.02)	7 (0.01)	3 (0.01)	2 (0.02)	9 (0.01)	9 (0.01)
	Total	290	612	330	114	620	726
	Allele frequency A/G	0.88/0.12	0.89/0.11	0.91/0.09	0.86/0.14	0.89/0.11	0.89/0.11
rs2835740	TT	187 (0.65)	366 (0.62)	217 (0.66)	74 (0.66)	404 (0.66)	440 (0.62)
	TC	86 (0.30)	206 (0.35)	90 (0.28)	31 (0.28)	176 (0.29)	237 (0.34)
	CC	13 (0.05)	24 (0.04)	19 (0.06)	7 (0.06)	32 (0.05)	31 (0.04)
	Total	286	596	326	112	612	708
	Allele frequency T/C	0.80/0.20	0.79/0.21	0.80/0.20	0.80/0.20	0.80/0.20	0.79/0.21
rs1137600	AA	124 (0.44)	276 (0.46)	164 (0.53)	50 (0.45)	288 (0.49)	326 (0.46)
	AG	124 (0.44)	261 (0.43)	107 (0.35)	46 (0.42)	231 (0.39)	307 (0.43)
	GG	32 (0.12)	69 (0.11)	37 (0.12)	14 (0.13)	69 (0.12)	83 (0.12)
	Total	280	606	308	110	588	716
	Allele frequency A/G	0.62/0.38	0.67/0.33	0.71/0.29	0.66/0.34	0.69/0.31	0.67/0.33
rs2835761	CC	203 (0.70)	422 (0.69)	232 (0.72)	84 (0.75)	435 (0.71)	506 (0.70)
	CT	82 (0.29)	176 (0.29)	85 (0.26)	27 (0.24)	167 (0.27)	203 (0.28)
	TT	4 (0.01)	11 (0.02)	6 (0.02)	1 (0.01)	10 (0.02)	12 (0.02)
	Total	289	609	323	112	612	721
	Allele frequency C/T	0.84/0.16	0.84/0.16	0.85/0.15	0.87/0.13	0.85/0.15	0.84/0.16
rs2835762	CC	216 (0.81)	467 (0.78)	243 (0.77)	82 (0.73)	459 (0.79)	549 (0.78)
	CT	49 (0.18)	119 (0.20)	69 (0.22)	27 (0.24)	118 (0.20)	146 (0.21)
	TT	3 (0.01)	9 (0.02)	4 (0.01)	3 (0.03)	7(0.01)	12 (0.01)
	Total	268	595	316	112	584	707
	Allele frequency C/T	0.90/0.10	0.88/0.12	0.88/0.12	0.85/0.15	0.89/0.11	0.88/0.12
rs2154545	GG	105 (0.37)	218 (0.36)	141 (0.43)	43 (0.38)	246 (0.40)	261 (0.36)
	GA	136 (0.47)	280 (0.46)	135 (0.42)	49 (0.44)	271 (0.44)	329 (0.46)
	AA	45 (0.16)	108 (0.18)	50 (0.15)	20 (0.18)	95 (0.16)	128 (0.18)
	Total	286	606	326	112	612	718
	Allele frequency G/A	0.60/0.40	0.59/0.41	0.64/0.36	0.60/0.40	0.62/0.38	0.59/0.41
rs8132976	AA	95 (0.34)	194 (0.33)	93 (0.29)	33 (0.30)	188 (0.31)	227 (0.32)
	AC	122 (0.43)	285 (0.48)	138 (0.43)	48 (0.43)	260 (0.43)	333 (0.47)
	CC	66 (0.23)	116 (0.19)	91 (0.28)	30 (0.27)	157 (0.26)	146 (0.21)
	Total	283	595	322	111	605	706
	Allele frequency A/C	0.55/0.45	0.57/0.43	0.50/0.50	0.51/0.49	0.53/0.47	0.56/0.44

Figures in parentheses indicate frequencies; p-values > 0.05 for all allelic and genotypic comparisons; p-values were not corrected for multiple comparisons.

**Table 2:** Haplotype association analysis between DYRK1A gene and AD

Haplotype block	Haplotype frequency	AD, control frequency	P value
ATACCG	0.329	0.347, 0.314	0.07
ACGCCA	0.202	0.190, 0.211	0.18
ATATCG	0.154	0.152, 0.155	0.82
ATACTG	0.115	0.112, 0.118	0.61
GTGCCA	0.109	0.107, 0.112	0.65
ATACCA	0.074	0.069, 0.077	0.43

Haplotype block consists of SNPs: rs11701483, rs2835740, rs1137600, rs2835761, rs2835762 and rs2154545. Rare haplotypes (total frequency < 0.05) were excluded from the analysis. P-values were not corrected for multiple comparisons.

## Discussion

In a series of 374 Japanese AD patients and 375 population-based controls, Kimura et al. [12] studied eight tagging SNPs (rs8126696, rs2251085, rs2835740, rs10470178, rs11701810, rs1024294, rs2835773 and rs2835774) located from 30 kb upstream of exon 1 to exon 13, observing a three times increased AD risk for carriers of the DYRK1A rs2835740 CC genotype (OR = 2.99, 95% CI = 1.72-5.19, p = 0.001), and haplotype analysis indicated that two haplotypes had significantly different frequencies between AD and controls. These authors showed that the expression of DYRK1A mRNA was elevated in the hippocampus of AD patients, coinciding with another report of increased DYRK1A immunoreactivity in the frontal cortex, entorhinal cortex and hippocampus of AD patients [4]. All the tagging SNPs analyzed in our study are located in a single block that does not cover the whole gene (captures 86% of DYRK1A genetic variability), but is the same haplotype block as described by Kimura et al. in their Japanese cohort. It could have been a great interest to identify possible DYRK1A genetic association using tagging SNPs located in haplotype blocks flanking the block illustrated in Figure 1; however, our main objective was to study the most strongly associated SNP rs2835740 (intron 3) of the original paper suggesting DYRK1A as a putative gene causing AD [12], and, in addition, we genotyped other SNPs in intron 3 (rs11701483, rs1137600 and rs2835761), intron 4 (rs2835762) and intron 10 (rs2154545 and rs8132976). In contrast with the findings of Kimura et al., we did not find any genetic association. Our failure to replicate the main finding of Kimura et al. could be caused by several factors. The possibility of a type 2 error (false-negative) must be taken in account, but we had enough power (98%) to detect an odds ratio of 1.5 at disease allele frequencies of approximately 0.15. Another possibility is genetic heterogeneity between our sample sets and those of the original study. While DYRK1A rs2835740 revealed evidence for association in Japanese samples, this SNP was not associated with AD in our Caucasian sample, and independent replication studies are needed in this gene to verify or refute the finding here and to extend it to other ethnic groups.

## Conclusion

Our negative findings in the Spanish population argue against the hypothesis that DYRK1A genetic variations are causally related to AD risk. Still, additional studies using different sets of patients and control subjects deserve further attention, since supporting evidence for association between DYRK1A gene and AD risk in the Japanese population exists.

## Competing interests

The authors declare that they have no competing interests.

## Authors' contributions

JLVH and ERR performed the genetic studies and reviewed critically the manuscript. PSJ performed the statistical analyses and reviewed critically the manuscript. IM, AP, AF, IS, FV, JB and MJB reviewed critically the manuscript. OC drafted the manuscript and contributed to its final version. All authors read and approved the final manuscript.

## Pre-publication history

The pre-publication history for this paper can be accessed here:

http://www.biomedcentral.com/1471-2350/10/129/prepub
